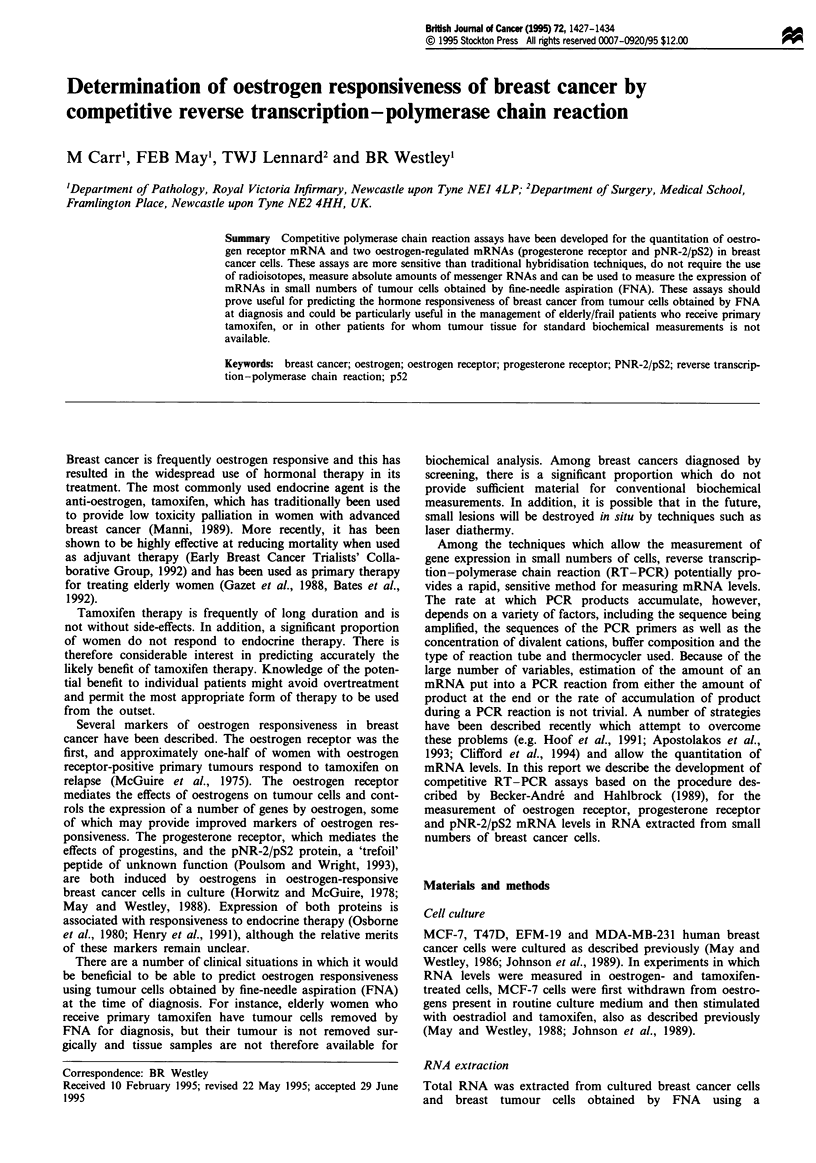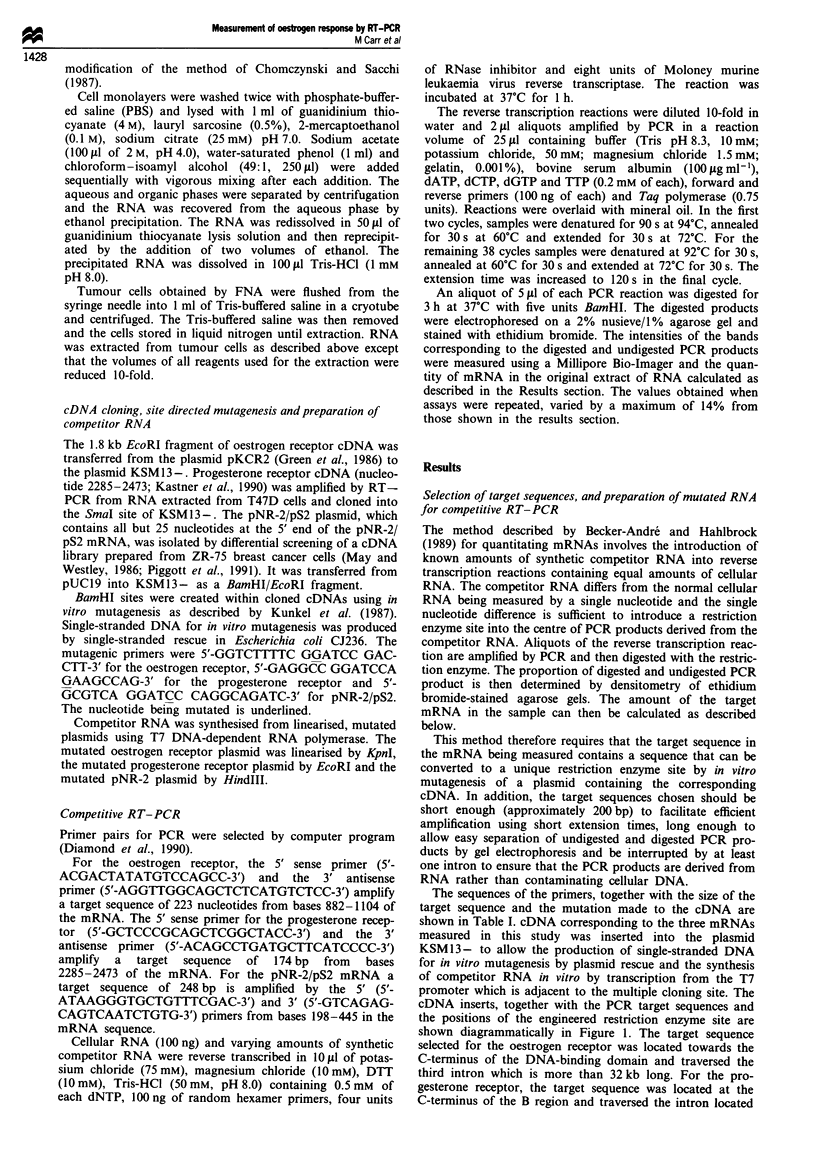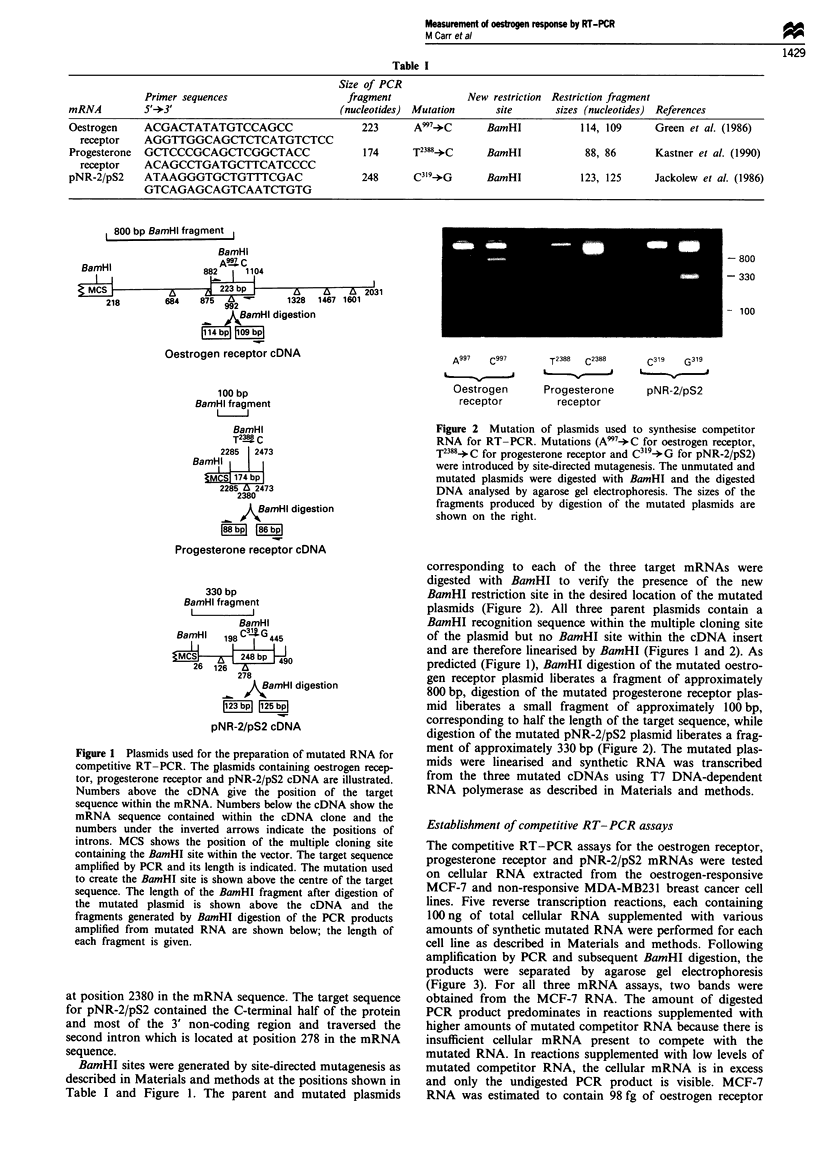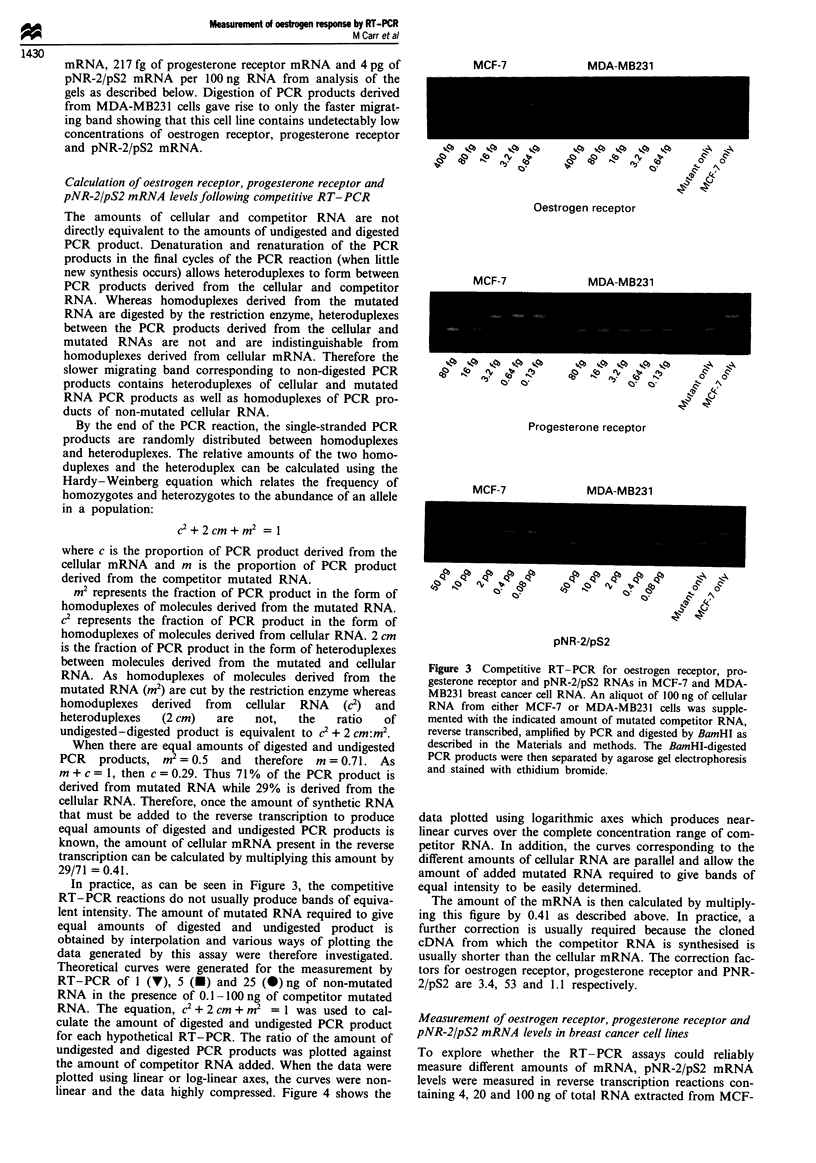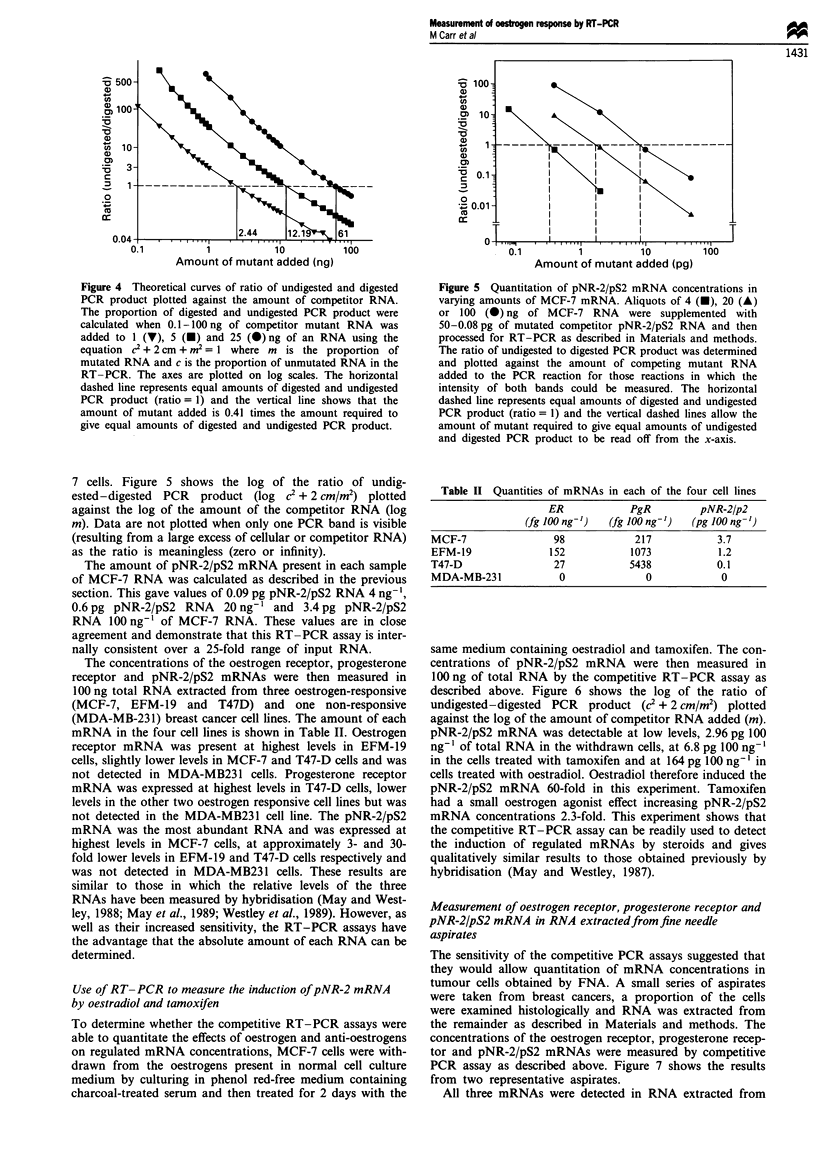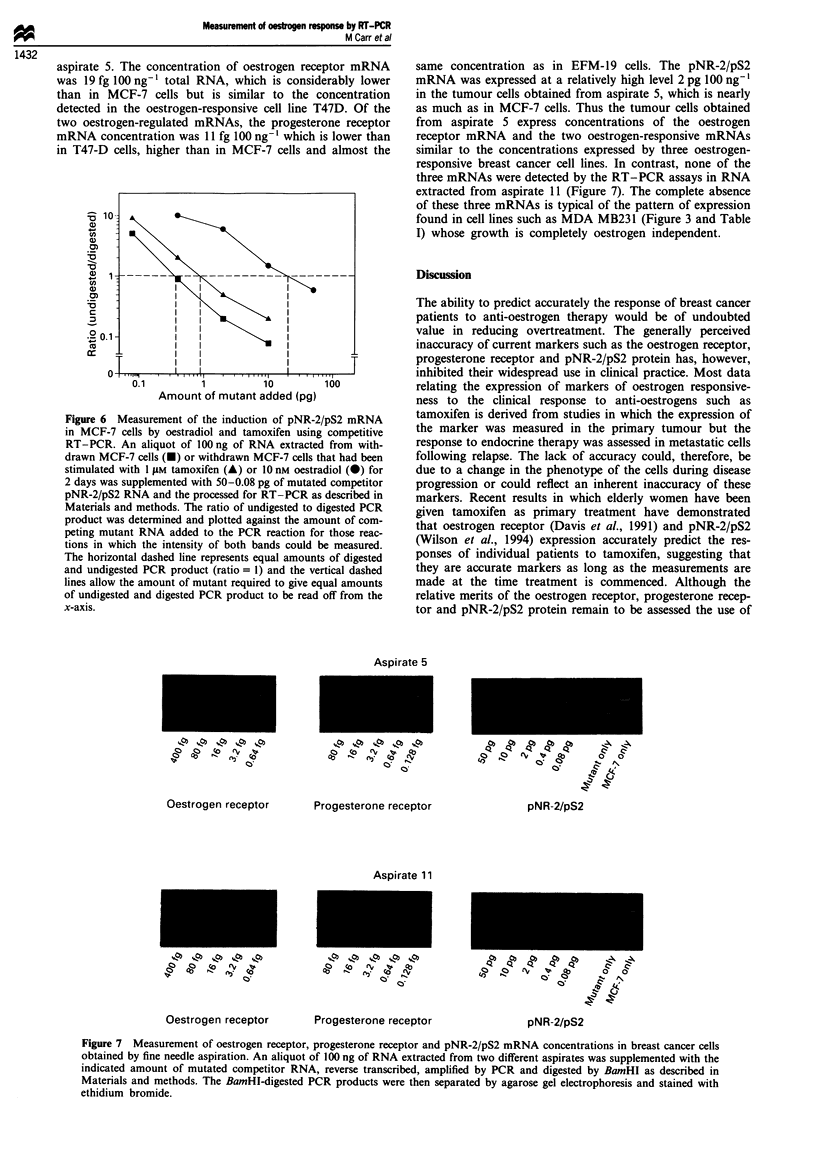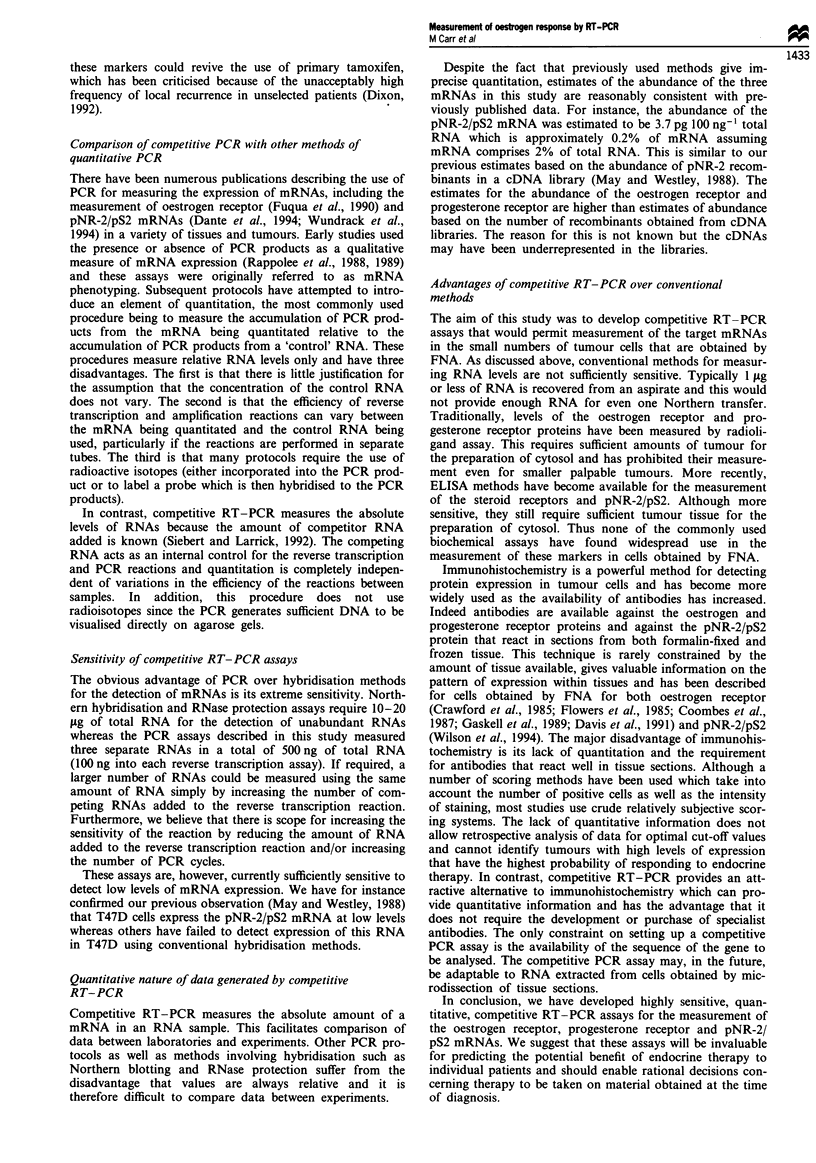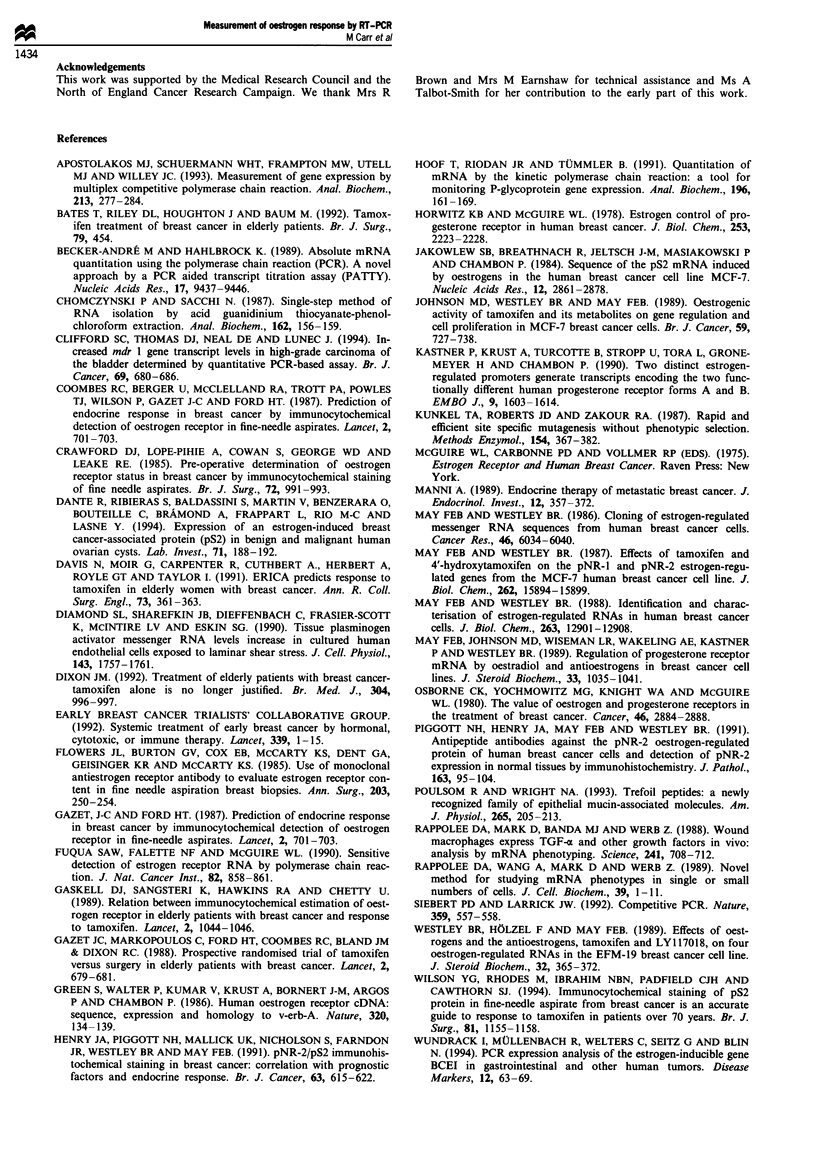# Determination of oestrogen responsiveness of breast cancer by competitive reverse transcription-polymerase chain reaction.

**DOI:** 10.1038/bjc.1995.525

**Published:** 1995-12

**Authors:** M. Carr, F. E. May, T. W. Lennard, B. R. Westley

**Affiliations:** Department of Pathology, Royal Victoria Infirmary, Newcastle upon Tyne, UK.

## Abstract

**Images:**


British Journal of Cancer (1995) 72, 1427–1434
© 1995 Stockton Press All rights reserved 0007–0920/95 $12.00

### Determination of oestrogen responsiveness of breast cancer by competitive reverse transcription–polymerase chain reaction

M Carr[1], FEB May[1], TWJ Lennard[2] and BR Westley[1]

[1]*Department of Pathology, Royal Victoria Infirmary, Newcastle upon Tyne NE1 4LP;* [2]*Department of Surgery, Medical School, Framlington Place, Newcastle upon Tyne NE2 4HH, UK.*

**Summary** Competitive polymerase chain reaction assays have been developed for the quantitation of oestrogen receptor mRNA and two oestrogen-regulated mRNAs (progesterone receptor and pNR-2/pS2) in breast cancer cells. These assays are more sensitive than traditional hybridisation techniques, do not require the use of radioisotopes, measure absolute amounts of messenger RNAs and can be used to measure the expression of mRNAs in small numbers of tumour cells obtained by fine-needle aspiration (FNA). These assays should prove useful for predicting the hormone responsiveness of breast cancer from tumour cells obtained by FNA at diagnosis and could be particularly useful in the management of elderly/frail patients who receive primary tamoxifen, or in other patients for whom tumour tissue for standard biochemical measurements is not available.

**Keywords:** breast cancer; oestrogen; oestrogen receptor; progesterone receptor; PNR-2/pS2; reverse transcription–polymerase chain reaction; p52

Breast cancer is frequently oestrogen responsive and this has resulted in the widespread use of hormonal therapy in its treatment. The most commonly used endocrine agent is the anti-oestrogen, tamoxifen, which has traditionally been used to provide low toxicity palliation in women with advanced breast cancer (Manni, 1989). More recently, it has been shown to be highly effective at reducing mortality when used as adjuvant therapy (Early Breast Cancer Trialists' Collaborative Group, 1992) and has been used as primary therapy for treating elderly women (Gazet *et al.*, 1988, Bates *et al.*, 1992).

Tamoxifen therapy is frequently of long duration and is not without side-effects. In addition, a significant proportion of women do not respond to endocrine therapy. There is therefore considerable interest in predicting accurately the likely benefit of tamoxifen therapy. Knowledge of the potential benefit to individual patients might avoid overtreatment and permit the most appropriate form of therapy to be used from the outset.

Several markers of oestrogen responsiveness in breast cancer have been described. The oestrogen receptor was the first, and approximately one-half of women with oestrogen receptor-positive primary tumours respond to tamoxifen on relapse (McGuire *et al.*, 1975). The oestrogen receptor mediates the effects of oestrogens on tumour cells and controls the expression of a number of genes by oestrogen, some of which may provide improved markers of oestrogen responsiveness. The progesterone receptor, which mediates the effects of progestins, and the pNR-2/pS2 protein, a 'trefoil' peptide of unknown function (Poulsom and Wright, 1993), are both induced by oestrogens in oestrogen-responsive breast cancer cells in culture (Horwitz and McGuire, 1978; May and Westley, 1988). Expression of both proteins is associated with responsiveness to endocrine therapy (Osborne *et al.*, 1980; Henry *et al.*, 1991), although the relative merits of these markers remain unclear.

There are a number of clinical situations in which it would be beneficial to be able to predict oestrogen responsiveness using tumour cells obtained by fine-needle aspiration (FNA) at the time of diagnosis. For instance, elderly women who receive primary tamoxifen have tumour cells removed by FNA for diagnosis, but their tumour is not removed surgically and tissue samples are not therefore available for biochemical analysis. Among breast cancers diagnosed by screening, there is a significant proportion which do not provide sufficient material for conventional biochemical measurements. In addition, it is possible that in the future, small lesions will be destroyed *in situ* by techniques such as laser diathermy.

Among the techniques which allow the measurement of gene expression in small numbers of cells, reverse transcription–polymerase chain reaction (RT–PCR) potentially provides a rapid, sensitive method for measuring mRNA levels. The rate at which PCR products accumulate, however, depends on a variety of factors, including the sequence being amplified, the sequences of the PCR primers as well as the concentration of divalent cations, buffer composition and the type of reaction tube and thermocycler used. Because of the large number of variables, estimation of the amount of an mRNA put into a PCR reaction from either the amount of product at the end or the rate of accumulation of product during a PCR reaction is not trivial. A number of strategies have been described recently which attempt to overcome these problems (e.g. Hoof *et al.*, 1991; Apostolakos *et al.*, 1993; Clifford *et al.*, 1994) and allow the quantitation of mRNA levels. In this report we describe the development of competitive RT–PCR assays based on the procedure described by Becker-André and Hahlbrock (1989), for the measurement of oestrogen receptor, progesterone receptor and pNR-2/pS2 mRNA levels in RNA extracted from small numbers of breast cancer cells.

### Materials and methods

### *Cell culture*

MCF-7, T47D, EFM-19 and MDA-MB-231 human breast cancer cells were cultured as described previously (May and Westley, 1986; Johnson *et al.*, 1989). In experiments in which RNA levels were measured in oestrogen- and tamoxifen-treated cells, MCF-7 cells were first withdrawn from oestrogens present in routine culture medium and then stimulated with oestradiol and tamoxifen, also as described previously (May and Westley, 1988; Johnson *et al.*, 1989).

### *RNA extraction*

Total RNA was extracted from cultured breast cancer cells and breast tumour cells obtained by FNA using a

Correspondence: BR Westley
Received 10 February 1995; revised 22 May 1995; accepted 29 June 1995

modification of the method of Chomczynski and Sacchi (1987).

Cell monolayers were washed twice with phosphate-buffered saline (PBS) and lysed with 1 ml of guanidinium thiocyanate (4 M), lauryl sarcosine (0.5%), 2-mercaptoethanol (0.1 M), sodium citrate (25 mM) pH 7.0. Sodium acetate (100 µl of 2 M, pH 4.0), water-saturated phenol (1 ml) and chloroform-isoamyl alcohol (49:1, 250 µl) were added sequentially with vigorous mixing after each addition. The aqueous and organic phases were separated by centrifugation and the RNA was recovered from the aqueous phase by ethanol precipitation. The RNA was redissolved in 50 µl of guanidinium thiocyanate lysis solution and then reprecipitated by the addition of two volumes of ethanol. The precipitated RNA was dissolved in 100 µl Tris-HCl (1 mM pH 8.0).

Tumour cells obtained by FNA were flushed from the syringe needle into 1 ml of Tris-buffered saline in a cryotube and centrifuged. The Tris-buffered saline was then removed and the cells stored in liquid nitrogen until extraction. RNA was extracted from tumour cells as described above except that the volumes of all reagents used for the extraction were reduced 10-fold.

### *cDNA cloning, site directed mutagenesis and preparation of competitor RNA*

The 1.8 kb *Eco*RI fragment of oestrogen receptor cDNA was transferred from the plasmid pKCR2 (Green *et al.*, 1986) to the plasmid KSM13−. Progesterone receptor cDNA (nucleotide 2285–2473; Kastner *et al.*, 1990) was amplified by RT—PCR from RNA extracted from T47D cells and cloned into the *Sma*I site of KSM13−. The pNR-2/pS2 plasmid, which contains all but 25 nucleotides at the 5′ end of the pNR-2/pS2 mRNA, was isolated by differential screening of a cDNA library prepared from ZR-75 breast cancer cells (May and Westley, 1986; Piggott *et al.*, 1991). It was transferred from pUC19 into KSM13− as a *Bam*HI/*Eco*RI fragment.

*Bam*HI sites were created within cloned cDNAs using *in vitro* mutagenesis as described by Kunkel *et al.* (1987). Single-stranded DNA for *in vitro* mutagenesis was produced by single-stranded rescue in *Escherichia coli* CJ236. The mutagenic primers were 5′-GGTCTTTTC GGATCC GACCTT-3′ for the oestrogen receptor, 5′-GAGGCC GGATCCA GAAGCCAG-3′ for the progesterone receptor and 5′-GCGTCA GGATCC CAGGCAGATC-3′ for pNR-2/pS2. The nucleotide being mutated is underlined.

Competitor RNA was synthesised from linearised, mutated plasmids using T7 DNA-dependent RNA polymerase. The mutated oestrogen receptor plasmid was linearised by *Kpn*I, the mutated progesterone receptor plasmid by *Eco*RI and the mutated pNR-2 plasmid by *Hin*dIII.

### *Competitive RT-PCR*

Primer pairs for PCR were selected by computer program (Diamond *et al.*, 1990).

For the oestrogen receptor, the 5′ sense primer (5′-ACGACTATATGTCCAGCC-3′) and the 3′ antisense primer (5′-AGGTTGGCAGCTCTCATGTCTCC-3′) amplify a target sequence of 223 nucleotides from bases 882–1104 of the mRNA. The 5′ sense primer for the progesterone receptor (5′-GCTCCCGCAGCTCGGCTACC-3′) and the 3′ antisense primer (5′-ACAGCCTGATGCTTCATCCCC-3′) amplify a target sequence of 174 bp from bases 2285–2473 of the mRNA. For the pNR-2/pS2 mRNA a target sequence of 248 bp is amplified by the 5′ (5′-ATAAGGGTGCTGTTTCGAC-3′) and 3′ (5′-GTCAGAGCAGTCAATCTGTG-3′) primers from bases 198–445 in the mRNA sequence.

Cellular RNA (100 ng) and varying amounts of synthetic competitor RNA were reverse transcribed in 10 µl of potassium chloride (75 mM), magnesium chloride (10 mM), DTT (10 mM), Tris-HCl (50 mM, pH 8.0) containing 0.5 mM of each dNTP, 100 ng of random hexamer primers, four units of RNase inhibitor and eight units of Moloney murine leukaemia virus reverse transcriptase. The reaction was incubated at 37°C for 1 h.

The reverse transcription reactions were diluted 10-fold in water and 2 µl aliquots amplified by PCR in a reaction volume of 25 µl containing buffer (Tris pH 8.3, 10 mM; potassium chloride, 50 mM; magnesium chloride 1.5 mM; gelatin, 0.001%), bovine serum albumin (100 $\mu g\ ml^{-1}$), dATP, dCTP, dGTP and TTP (0.2 mM of each), forward and reverse primers (100 ng of each) and *Taq* polymerase (0.75 units). Reactions were overlaid with mineral oil. In the first two cycles, samples were denatured for 90 s at 94°C, annealed for 30 s at 60°C and extended for 30 s at 72°C. For the remaining 38 cycles samples were denatured at 92°C for 30 s, annealed at 60°C for 30 s and extended at 72°C for 30 s. The extension time was increased to 120 s in the final cycle.

An aliquot of 5 µl of each PCR reaction was digested for 3 h at 37°C with five units *Bam*HI. The digested products were electrophoresed on a 2% nusieve/1% agarose gel and stained with ethidium bromide. The intensities of the bands corresponding to the digested and undigested PCR products were measured using a Millipore Bio-Imager and the quantity of mRNA in the original extract of RNA calculated as described in the Results section. The values obtained when assays were repeated, varied by a maximum of 14% from those shown in the results section.

### Results

### *Selection of target sequences, and preparation of mutated RNA for competitive RT-PCR*

The method described by Becker-André and Hahlbrock (1989) for quantitating mRNAs involves the introduction of known amounts of synthetic competitor RNA into reverse transcription reactions containing equal amounts of cellular RNA. The competitor RNA differs from the normal cellular RNA being measured by a single nucleotide and the single nucleotide difference is sufficient to introduce a restriction enzyme site into the centre of PCR products derived from the competitor RNA. Aliquots of the reverse transcription reaction are amplified by PCR and then digested with the restriction enzyme. The proportion of digested and undigested PCR product is then determined by densitometry of ethidium bromide-stained agarose gels. The amount of the target mRNA in the sample can then be calculated as described below.

This method therefore requires that the target sequence in the mRNA being measured contains a sequence that can be converted to a unique restriction enzyme site by *in vitro* mutagenesis of a plasmid containing the corresponding cDNA. In addition, the target sequences chosen should be short enough (approximately 200 bp) to facilitate efficient amplification using short extension times, long enough to allow easy separation of undigested and digested PCR products by gel electrophoresis and be interrupted by at least one intron to ensure that the PCR products are derived from RNA rather than contaminating cellular DNA.

The sequences of the primers, together with the size of the target sequence and the mutation made to the cDNA are shown in Table I. cDNA corresponding to the three mRNAs measured in this study was inserted into the plasmid KSM13− to allow the production of single-stranded DNA for *in vitro* mutagenesis by plasmid rescue and the synthesis of competitor RNA *in vitro* by transcription from the T7 promoter which is adjacent to the multiple cloning site. The cDNA inserts, together with the PCR target sequences and the positions of the engineered restriction enzyme site are shown diagrammatically in Figure 1. The target sequence selected for the oestrogen receptor was located towards the C-terminus of the DNA-binding domain and traversed the third intron which is more than 32 kb long. For the progesterone receptor, the target sequence was located at the C-terminus of the B region and traversed the intron located

**Table I**

| mRNA | Primer sequences 5'→3' | Size of PCR fragment (nucleotides) | Mutation | New restriction site | Restriction fragment sizes (nucleotides) | References |
|---|---|---|---|---|---|---|
| Oestrogen receptor | ACGACTATATGTCCAGCC<br>AGGTTGGCAGCTCTCATGTCTCC | 223 | $A^{997}$→C | *Bam*HI | 114, 109 | Green *et al.* (1986) |
| Progesterone receptor | GCTCCCGCAGCTCGGCTACC<br>ACAGCCTGATGCTTCATCCCC | 174 | $T^{2388}$→C | *Bam*HI | 88, 86 | Kastner *et al.* (1990) |
| pNR-2/pS2 | ATAAGGGTGCTGTTTCGAC<br>GTCAGAGCAGTCAATCTGTG | 248 | $C^{319}$→G | *Bam*HI | 123, 125 | Jackolew *et al.* (1986) |

**Figure 1** Plasmids used for the preparation of mutated RNA for competitive RT-PCR. The plasmids containing oestrogen receptor, progesterone receptor and pNR-2/pS2 cDNA are illustrated. Numbers above the cDNA give the position of the target sequence within the mRNA. Numbers below the cDNA show the mRNA sequence contained within the cDNA clone and the numbers under the inverted arrows indicate the positions of introns. MCS shows the position of the multiple cloning site containing the *Bam*HI site within the vector. The target sequence amplified by PCR and its length is indicated. The mutation used to create the *Bam*HI site is shown above the centre of the target sequence. The length of the *Bam*HI fragment after digestion of the mutated plasmid is shown above the cDNA and the fragments generated by *Bam*HI digestion of the PCR products amplified from mutated RNA are shown below; the length of each fragment is given.

**Figure 2** Mutation of plasmids used to synthesise competitor RNA for RT-PCR. Mutations ($A^{997}$→C for oestrogen receptor, $T^{2388}$→C for progesterone receptor and $C^{319}$→G for pNR-2/pS2) were introduced by site-directed mutagenesis. The unmutated and mutated plasmids were digested with *Bam*HI and the digested DNA analysed by agarose gel electrophoresis. The sizes of the fragments produced by digestion of the mutated plasmids are shown on the right.

at position 2380 in the mRNA sequence. The target sequence for pNR-2/pS2 contained the C-terminal half of the protein and most of the 3' non-coding region and traversed the second intron which is located at position 278 in the mRNA sequence.

*Bam*HI sites were generated by site-directed mutagenesis as described in Materials and methods at the positions shown in Table I and Figure 1. The parent and mutated plasmids corresponding to each of the three target mRNAs were digested with *Bam*HI to verify the presence of the new *Bam*HI restriction site in the desired location of the mutated plasmids (Figure 2). All three parent plasmids contain a *Bam*HI recognition sequence within the multiple cloning site of the plasmid but no *Bam*HI site within the cDNA insert and are therefore linearised by *Bam*HI (Figures 1 and 2). As predicted (Figure 1), *Bam*HI digestion of the mutated oestrogen receptor plasmid liberates a fragment of approximately 800 bp, digestion of the mutated progesterone receptor plasmid liberates a small fragment of approximately 100 bp, corresponding to half the length of the target sequence, while digestion of the mutated pNR-2/pS2 plasmid liberates a fragment of approximately 330 bp (Figure 2). The mutated plasmids were linearised and synthetic RNA was transcribed from the three mutated cDNAs using T7 DNA-dependent RNA polymerase as described in Materials and methods.

### *Establishment of competitive RT-PCR assays*

The competitive RT-PCR assays for the oestrogen receptor, progesterone receptor and pNR-2/pS2 mRNAs were tested on cellular RNA extracted from the oestrogen-responsive MCF-7 and non-responsive MDA-MB231 breast cancer cell lines. Five reverse transcription reactions, each containing 100 ng of total cellular RNA supplemented with various amounts of synthetic mutated RNA were performed for each cell line as described in Materials and methods. Following amplification by PCR and subsequent *Bam*HI digestion, the products were separated by agarose gel electrophoresis (Figure 3). For all three mRNA assays, two bands were obtained from the MCF-7 RNA. The amount of digested PCR product predominates in reactions supplemented with higher amounts of mutated competitor RNA because there is insufficient cellular mRNA present to compete with the mutated RNA. In reactions supplemented with low levels of mutated competitor RNA, the cellular mRNA is in excess and only the undigested PCR product is visible. MCF-7 RNA was estimated to contain 98 fg of oestrogen receptor mRNA, 217 fg of progesterone receptor mRNA and 4 pg of pNR-2/pS2 mRNA per 100 ng RNA from analysis of the gels as described below. Digestion of PCR products derived from MDA-MB231 cells gave rise to only the faster migrating band showing that this cell line contains undetectably low concentrations of oestrogen receptor, progesterone receptor and pNR-2/pS2 mRNA.

*Calculation of oestrogen receptor, progesterone receptor and pNR-2/pS2 mRNA levels following competitive RT-PCR*

The amounts of cellular and competitor RNA are not directly equivalent to the amounts of undigested and digested PCR product. Denaturation and renaturation of the PCR products in the final cycles of the PCR reaction (when little new synthesis occurs) allows heteroduplexes to form between PCR products derived from the cellular and competitor RNA. Whereas homoduplexes derived from the mutated RNA are digested by the restriction enzyme, heteroduplexes between the PCR products derived from the cellular and mutated RNAs are not and are indistinguishable from homoduplexes derived from cellular mRNA. Therefore the slower migrating band corresponding to non-digested PCR products contains heteroduplexes of cellular and mutated RNA PCR products as well as homoduplexes of PCR products of non-mutated cellular RNA.

By the end of the PCR reaction, the single-stranded PCR products are randomly distributed between homoduplexes and heteroduplexes. The relative amounts of the two homoduplexes and the heteroduplex can be calculated using the Hardy-Weinberg equation which relates the frequency of homozygotes and heterozygotes to the abundance of an allele in a population:

$$c^2 + 2\,cm + m^2 = 1$$

where $c$ is the proportion of PCR product derived from the cellular mRNA and $m$ is the proportion of PCR product derived from the competitor mutated RNA.

$m^2$ represents the fraction of PCR product in the form of homoduplexes of molecules derived from the mutated RNA. $c^2$ represents the fraction of PCR product in the form of homoduplexes of molecules derived from cellular RNA. $2\,cm$ is the fraction of PCR product in the form of heteroduplexes between molecules derived from the mutated and cellular RNA. As homoduplexes of molecules derived from the mutated RNA ($m^2$) are cut by the restriction enzyme whereas homoduplexes derived from cellular RNA ($c^2$) and heteroduplexes ($2\,cm$) are not, the ratio of undigested-digested product is equivalent to $c^2 + 2\,cm{:}m^2$.

When there are equal amounts of digested and undigested PCR products, $m^2 = 0.5$ and therefore $m = 0.71$. As $m + c = 1$, then $c = 0.29$. Thus 71% of the PCR product is derived from mutated RNA while 29% is derived from the cellular RNA. Therefore, once the amount of synthetic RNA that must be added to the reverse transcription to produce equal amounts of digested and undigested PCR products is known, the amount of cellular mRNA present in the reverse transcription can be calculated by multiplying this amount by $29/71 = 0.41$.

In practice, as can be seen in Figure 3, the competitive RT-PCR reactions do not usually produce bands of equivalent intensity. The amount of mutated RNA required to give equal amounts of digested and undigested product is obtained by interpolation and various ways of plotting the data generated by this assay were therefore investigated. Theoretical curves were generated for the measurement by RT-PCR of 1 (▼), 5 (■) and 25 (●) ng of non-mutated RNA in the presence of 0.1-100 ng of competitor mutated RNA. The equation, $c^2 + 2\,cm + m^2 = 1$ was used to calculate the amount of digested and undigested PCR product for each hypothetical RT-PCR. The ratio of the amount of undigested and digested PCR products was plotted against the amount of competitor RNA added. When the data were plotted using linear or log-linear axes, the curves were non-linear and the data highly compressed. Figure 4 shows the

**Figure 3** Competitive RT-PCR for oestrogen receptor, progesterone receptor and pNR-2/pS2 RNAs in MCF-7 and MDA-MB231 breast cancer cell RNA. An aliquot of 100 ng of cellular RNA from either MCF-7 or MDA-MB231 cells was supplemented with the indicated amount of mutated competitor RNA, reverse transcribed, amplified by PCR and digested by *Bam*HI as described in the Materials and methods. The *Bam*HI-digested PCR products were then separated by agarose gel electrophoresis and stained with ethidium bromide.

data plotted using logarithmic axes which produces near-linear curves over the complete concentration range of competitor RNA. In addition, the curves corresponding to the different amounts of cellular RNA are parallel and allow the amount of added mutated RNA required to give bands of equal intensity to be easily determined.

The amount of the mRNA is then calculated by multiplying this figure by 0.41 as described above. In practice, a further correction is usually required because the cloned cDNA from which the competitor RNA is synthesised is usually shorter than the cellular mRNA. The correction factors for oestrogen receptor, progesterone receptor and PNR-2/pS2 are 3.4, 53 and 1.1 respectively.

*Measurement of oestrogen receptor, progesterone receptor and pNR-2/pS2 mRNA levels in breast cancer cell lines*

To explore whether the RT-PCR assays could reliably measure different amounts of mRNA, pNR-2/pS2 mRNA levels were measured in reverse transcription reactions containing 4, 20 and 100 ng of total RNA extracted from MCF-

**Figure 4** Theoretical curves of ratio of undigested and digested PCR product plotted against the amount of competitor RNA. The proportion of digested and undigested PCR product were calculated when 0.1–100 ng of competitor mutant RNA was added to 1 (▼), 5 (■) and 25 (●) ng of an RNA using the equation $c^2 + 2\,\mathrm{cm} + m^2 = 1$ where $m$ is the proportion of mutated RNA and $c$ is the proportion of unmutated RNA in the RT–PCR. The axes are plotted on log scales. The horizontal dashed line represents equal amounts of digested and undigested PCR product (ratio = 1) and the vertical line shows that the amount of mutant added is 0.41 times the amount required to give equal amounts of digested and undigested PCR product.

**Figure 5** Quantitation of pNR-2/pS2 mRNA concentrations in varying amounts of MCF-7 mRNA. Aliquots of 4 (■), 20 (▲) or 100 (●) ng of MCF-7 RNA were supplemented with 50–0.08 pg of mutated competitor pNR-2/pS2 RNA and then processed for RT–PCR as described in Materials and methods. The ratio of undigested to digested PCR product was determined and plotted against the amount of competing mutant RNA added to the PCR reaction for those reactions in which the intensity of both bands could be measured. The horizontal dashed line represents equal amounts of digested and undigested PCR product (ratio = 1) and the vertical dashed lines allow the amount of mutant required to give equal amounts of undigested and digested PCR product to be read off from the $x$-axis.

7 cells. Figure 5 shows the log of the ratio of undigested–digested PCR product (log $c^2 + 2\,cm/m^2$) plotted against the log of the amount of the competitor RNA (log $m$). Data are not plotted when only one PCR band is visible (resulting from a large excess of cellular or competitor RNA) as the ratio is meaningless (zero or infinity).

The amount of pNR-2/pS2 mRNA present in each sample of MCF-7 RNA was calculated as described in the previous section. This gave values of 0.09 pg pNR-2/pS2 RNA 4 $\mathrm{ng}^{-1}$, 0.6 pg pNR-2/pS2 RNA 20 $\mathrm{ng}^{-1}$ and 3.4 pg pNR-2/pS2 RNA 100 $\mathrm{ng}^{-1}$ of MCF-7 RNA. These values are in close agreement and demonstrate that this RT–PCR assay is internally consistent over a 25-fold range of input RNA.

The concentrations of the oestrogen receptor, progesterone receptor and pNR-2/pS2 mRNAs were then measured in 100 ng total RNA extracted from three oestrogen-responsive (MCF-7, EFM-19 and T47D) and one non-responsive (MDA-MB-231) breast cancer cell lines. The amount of each mRNA in the four cell lines is shown in Table II. Oestrogen receptor mRNA was present at highest levels in EFM-19 cells, slightly lower levels in MCF-7 and T47-D cells and was not detected in MDA-MB231 cells. Progesterone receptor mRNA was expressed at highest levels in T47-D cells, lower levels in the other two oestrogen responsive cell lines but was not detected in the MDA-MB231 cell line. The pNR-2/pS2 mRNA was the most abundant RNA and was expressed at highest levels in MCF-7 cells, at approximately 3- and 30-fold lower levels in EFM-19 and T47-D cells respectively and was not detected in MDA-MB231 cells. These results are similar to those in which the relative levels of the three RNAs have been measured by hybridisation (May and Westley, 1988; May *et al.*, 1989; Westley *et al.*, 1989). However, as well as their increased sensitivity, the RT–PCR assays have the advantage that the absolute amount of each RNA can be determined.

*Use of RT–PCR to measure the induction of pNR-2 mRNA by oestradiol and tamoxifen*

To determine whether the competitive RT–PCR assays were able to quantitate the effects of oestrogen and anti-oestrogens on regulated mRNA concentrations, MCF-7 cells were withdrawn from the oestrogens present in normal cell culture medium by culturing in phenol red-free medium containing charcoal-treated serum and then treated for 2 days with the same medium containing oestradiol and tamoxifen. The concentrations of pNR-2/pS2 mRNA were then measured in 100 ng of total RNA by the competitive RT–PCR assay as described above. Figure 6 shows the log of the ratio of undigested–digested PCR product ($c^2 + 2\,cm/m^2$) plotted against the log of the amount of competitor RNA added ($m$). pNR-2/pS2 mRNA was detectable at low levels, 2.96 pg 100 $\mathrm{ng}^{-1}$ of total RNA in the withdrawn cells, at 6.8 pg 100 $\mathrm{ng}^{-1}$ in the cells treated with tamoxifen and at 164 pg 100 $\mathrm{ng}^{-1}$ in cells treated with oestradiol. Oestradiol therefore induced the pNR-2/pS2 mRNA 60-fold in this experiment. Tamoxifen had a small oestrogen agonist effect increasing pNR-2/pS2 mRNA concentrations 2.3-fold. This experiment shows that the competitive RT–PCR assay can be readily used to detect the induction of regulated mRNAs by steroids and gives qualitatively similar results to those obtained previously by hybridisation (May and Westley, 1987).

**Table II** Quantities of mRNAs in each of the four cell lines

| | *ER* (*fg 100 ng*$^{-1}$) | *PgR* (*fg 100 ng*$^{-1}$) | *pNR-2/p2* (*pg 100 ng*$^{-1}$) |
|---|---|---|---|
| MCF-7 | 98 | 217 | 3.7 |
| EFM-19 | 152 | 1073 | 1.2 |
| T47-D | 27 | 5438 | 0.1 |
| MDA-MB-231 | 0 | 0 | 0 |

*Measurement of oestrogen receptor, progesterone receptor and pNR-2/pS2 mRNA in RNA extracted from fine needle aspirates*

The sensitivity of the competitive PCR assays suggested that they would allow quantitation of mRNA concentrations in tumour cells obtained by FNA. A small series of aspirates were taken from breast cancers, a proportion of the cells were examined histologically and RNA was extracted from the remainder as described in Materials and methods. The concentrations of the oestrogen receptor, progesterone receptor and pNR-2/pS2 mRNAs were measured by competitive PCR assay as described above. Figure 7 shows the results from two representative aspirates.

All three mRNAs were detected in RNA extracted from aspirate 5. The concentration of oestrogen receptor mRNA was 19 fg 100 ng$^{-1}$ total RNA, which is considerably lower than in MCF-7 cells but is similar to the concentration detected in the oestrogen-responsive cell line T47D. Of the two oestrogen-regulated mRNAs, the progesterone receptor mRNA concentration was 11 fg 100 ng$^{-1}$ which is lower than in T47-D cells, higher than in MCF-7 cells and almost the

**Figure 6** Measurement of the induction of pNR-2/pS2 mRNA in MCF-7 cells by oestradiol and tamoxifen using competitive RT-PCR. An aliquot of 100 ng of RNA extracted from withdrawn MCF-7 cells (■) or withdrawn MCF-7 cells that had been stimulated with 1 μM tamoxifen (▲) or 10 nM oestradiol (●) for 2 days was supplemented with 50–0.08 pg of mutated competitor pNR-2/pS2 RNA and the processed for RT-PCR as described in Materials and methods. The ratio of undigested to digested PCR product was determined and plotted against the amount of competing mutant RNA added to the PCR reaction for those reactions in which the intensity of both bands could be measured. The horizontal dashed line represents equal amounts of digested and undigested PCR product (ratio = 1) and the vertical dashed lines allow the amount of mutant required to give equal amounts of undigested and digested PCR product to be read off from the *x*-axis.

same concentration as in EFM-19 cells. The pNR-2/pS2 mRNA was expressed at a relatively high level 2 pg 100 ng$^{-1}$ in the tumour cells obtained from aspirate 5, which is nearly as much as in MCF-7 cells. Thus the tumour cells obtained from aspirate 5 express concentrations of the oestrogen receptor mRNA and the two oestrogen-responsive mRNAs similar to the concentrations expressed by three oestrogen-responsive breast cancer cell lines. In contrast, none of the three mRNAs were detected by the RT–PCR assays in RNA extracted from aspirate 11 (Figure 7). The complete absence of these three mRNAs is typical of the pattern of expression found in cell lines such as MDA MB231 (Figure 3 and Table I) whose growth is completely oestrogen independent.

### Discussion

The ability to predict accurately the response of breast cancer patients to anti-oestrogen therapy would be of undoubted value in reducing overtreatment. The generally perceived inaccuracy of current markers such as the oestrogen receptor, progesterone receptor and pNR-2/pS2 protein has, however, inhibited their widespread use in clinical practice. Most data relating the expression of markers of oestrogen responsiveness to the clinical response to anti-oestrogens such as tamoxifen is derived from studies in which the expression of the marker was measured in the primary tumour but the response to endocrine therapy was assessed in metastatic cells following relapse. The lack of accuracy could, therefore, be due to a change in the phenotype of the cells during disease progression or could reflect an inherent inaccuracy of these markers. Recent results in which elderly women have been given tamoxifen as primary treatment have demonstrated that oestrogen receptor (Davis *et al.*, 1991) and pNR-2/pS2 (Wilson *et al.*, 1994) expression accurately predict the responses of individual patients to tamoxifen, suggesting that they are accurate markers as long as the measurements are made at the time treatment is commenced. Although the relative merits of the oestrogen receptor, progesterone receptor and pNR-2/pS2 protein remain to be assessed the use of

**Figure 7** Measurement of oestrogen receptor, progesterone receptor and pNR-2/pS2 mRNA concentrations in breast cancer cells obtained by fine needle aspiration. An aliquot of 100 ng of RNA extracted from two different aspirates was supplemented with the indicated amount of mutated competitor RNA, reverse transcribed, amplified by PCR and digested by *Bam*HI as described in Materials and methods. The *Bam*HI-digested PCR products were then separated by agarose gel electrophoresis and stained with ethidium bromide.

these markers could revive the use of primary tamoxifen, which has been criticised because of the unacceptably high frequency of local recurrence in unselected patients (Dixon, 1992).

*Comparison of competitive PCR with other methods of quantitative PCR*

There have been numerous publications describing the use of PCR for measuring the expression of mRNAs, including the measurement of oestrogen receptor (Fuqua *et al.*, 1990) and pNR-2/pS2 mRNAs (Dante *et al.*, 1994; Wundrack *et al.*, 1994) in a variety of tissues and tumours. Early studies used the presence or absence of PCR products as a qualitative measure of mRNA expression (Rappolee *et al.*, 1988, 1989) and these assays were originally referred to as mRNA phenotyping. Subsequent protocols have attempted to introduce an element of quantitation, the most commonly used procedure being to measure the accumulation of PCR products from the mRNA being quantitated relative to the accumulation of PCR products from a 'control' RNA. These procedures measure relative RNA levels only and have three disadvantages. The first is that there is little justification for the assumption that the concentration of the control RNA does not vary. The second is that the efficiency of reverse transcription and amplification reactions can vary between the mRNA being quantitated and the control RNA being used, particularly if the reactions are performed in separate tubes. The third is that many protocols require the use of radioactive isotopes (either incorporated into the PCR product or to label a probe which is then hybridised to the PCR products).

In contrast, competitive RT–PCR measures the absolute levels of RNAs because the amount of competitor RNA added is known (Siebert and Larrick, 1992). The competing RNA acts as an internal control for the reverse transcription and PCR reactions and quantitation is completely independent of variations in the efficiency of the reactions between samples. In addition, this procedure does not use radioisotopes since the PCR generates sufficient DNA to be visualised directly on agarose gels.

*Sensitivity of competitive RT–PCR assays*

The obvious advantage of PCR over hybridisation methods for the detection of mRNAs is its extreme sensitivity. Northern hybridisation and RNase protection assays require 10–20 μg of total RNA for the detection of unabundant RNAs whereas the PCR assays described in this study measured three separate RNAs in a total of 500 ng of total RNA (100 ng into each reverse transcription assay). If required, a larger number of RNAs could be measured using the same amount of RNA simply by increasing the number of competing RNAs added to the reverse transcription reaction. Furthermore, we believe that there is scope for increasing the sensitivity of the reaction by reducing the amount of RNA added to the reverse transcription reaction and/or increasing the number of PCR cycles.

These assays are, however, currently sufficiently sensitive to detect low levels of mRNA expression. We have for instance confirmed our previous observation (May and Westley, 1988) that T47D cells express the pNR-2/pS2 mRNA at low levels whereas others have failed to detect expression of this RNA in T47D using conventional hybridisation methods.

*Quantitative nature of data generated by competitive RT–PCR*

Competitive RT–PCR measures the absolute amount of a mRNA in an RNA sample. This facilitates comparison of data between laboratories and experiments. Other PCR protocols as well as methods involving hybridisation such as Northern blotting and RNase protection suffer from the disadvantage that values are always relative and it is therefore difficult to compare data between experiments.

Despite the fact that previously used methods give imprecise quantitation, estimates of the abundance of the three mRNAs in this study are reasonably consistent with previously published data. For instance, the abundance of the pNR-2/pS2 mRNA was estimated to be 3.7 pg 100 $ng^{-1}$ total RNA which is approximately 0.2% of mRNA assuming mRNA comprises 2% of total RNA. This is similar to our previous estimates based on the abundance of pNR-2 recombinants in a cDNA library (May and Westley, 1988). The estimates for the abundance of the oestrogen receptor and progesterone receptor are higher than estimates of abundance based on the number of recombinants obtained from cDNA libraries. The reason for this is not known but the cDNAs may have been underrepresented in the libraries.

*Advantages of competitive RT–PCR over conventional methods*

The aim of this study was to develop competitive RT–PCR assays that would permit measurement of the target mRNAs in the small numbers of tumour cells that are obtained by FNA. As discussed above, conventional methods for measuring RNA levels are not sufficiently sensitive. Typically 1 μg or less of RNA is recovered from an aspirate and this would not provide enough RNA for even one Northern transfer. Traditionally, levels of the oestrogen receptor and progesterone receptor proteins have been measured by radioligand assay. This requires sufficient amounts of tumour for the preparation of cytosol and has prohibited their measurement even for smaller palpable tumours. More recently, ELISA methods have become available for the measurement of the steroid receptors and pNR-2/pS2. Although more sensitive, they still require sufficient tumour tissue for the preparation of cytosol. Thus none of the commonly used biochemical assays have found widespread use in the measurement of these markers in cells obtained by FNA.

Immunohistochemistry is a powerful method for detecting protein expression in tumour cells and has become more widely used as the availability of antibodies has increased. Indeed antibodies are available against the oestrogen and progesterone receptor proteins and against the pNR-2/pS2 protein that react in sections from both formalin-fixed and frozen tissue. This technique is rarely constrained by the amount of tissue available, gives valuable information on the pattern of expression within tissues and has been described for cells obtained by FNA for both oestrogen receptor (Crawford *et al.*, 1985; Flowers *et al.*, 1985; Coombes *et al.*, 1987; Gaskell *et al.*, 1989; Davis *et al.*, 1991) and pNR-2/pS2 (Wilson *et al.*, 1994). The major disadvantage of immunohistochemistry is its lack of quantitation and the requirement for antibodies that react well in tissue sections. Although a number of scoring methods have been used which take into account the number of positive cells as well as the intensity of staining, most studies use crude relatively subjective scoring systems. The lack of quantitative information does not allow retrospective analysis of data for optimal cut-off values and cannot identify tumours with high levels of expression that have the highest probability of responding to endocrine therapy. In contrast, competitive RT–PCR provides an attractive alternative to immunohistochemistry which can provide quantitative information and has the advantage that it does not require the development or purchase of specialist antibodies. The only constraint on setting up a competitive PCR assay is the availability of the sequence of the gene to be analysed. The competitive PCR assay may, in the future, be adaptable to RNA extracted from cells obtained by microdissection of tissue sections.

In conclusion, we have developed highly sensitive, quantitative, competitive RT–PCR assays for the measurement of the oestrogen receptor, progesterone receptor and pNR-2/pS2 mRNAs. We suggest that these assays will be invaluable for predicting the potential benefit of endocrine therapy to individual patients and should enable rational decisions concerning therapy to be taken on material obtained at the time of diagnosis.

**Acknowledgements**
This work was supported by the Medical Research Council and the North of England Cancer Research Campaign. We thank Mrs R Brown and Mrs M Earnshaw for technical assistance and Ms A Talbot-Smith for her contribution to the early part of this work.

**References**

APOSTOLAKOS MJ, SCHUERMANN WHT, FRAMPTON MW, UTELL MJ AND WILLEY JC. (1993). Measurement of gene expression by multiplex competitive polymerase chain reaction. *Anal. Biochem.*, **213**, 277-284.

BATES T, RILEY DL, HOUGHTON J AND BAUM M. (1992). Tamoxifen treatment of breast cancer in elderly patients. *Br. J. Surg.*, **79**, 454.

BECKER-ANDRÉ M AND HAHLBROCK K. (1989). Absolute mRNA quantitation using the polymerase chain reaction (PCR). A novel approach by a PCR aided transcript titration assay (PATTY). *Nucleic Acids Res.*, **17**, 9437-9446.

CHOMCZYNSKI P AND SACCHI N. (1987). Single-step method of RNA isolation by acid guanidinium thiocyanate-phenol-chloroform extraction. *Anal. Biochem.*, **162**, 156-159.

CLIFFORD SC, THOMAS DJ, NEAL DE AND LUNEC J. (1994). Increased *mdr* 1 gene transcript levels in high-grade carcinoma of the bladder determined by quantitative PCR-based assay. *Br. J. Cancer*, **69**, 680-686.

COOMBES RC, BERGER U, MCCLELLAND RA, TROTT PA, POWLES TJ, WILSON P, GAZET J-C AND FORD HT. (1987). Prediction of endocrine response in breast cancer by immunocytochemical detection of oestrogen receptor in fine-needle aspirates. *Lancet*, **2**, 701-703.

CRAWFORD DJ, LOPE-PIHIE A, COWAN S, GEORGE WD AND LEAKE RE. (1985). Pre-operative determination of oestrogen receptor status in breast cancer by immunocytochemical staining of fine needle aspirates. *Br. J. Surg.*, **72**, 991-993.

DANTE R, RIBIERAS S, BALDASSINI S, MARTIN V, BENZERARA O, BOUTEILLE C, BRÂMOND A, FRAPPART L, RIO M-C AND LASNE Y. (1994). Expression of an estrogen-induced breast cancer-associated protein (pS2) in benign and malignant human ovarian cysts. *Lab. Invest.*, **71**, 188-192.

DAVIS N, MOIR G, CARPENTER R, CUTHBERT A., HERBERT A, ROYLE GT AND TAYLOR I. (1991). ERICA predicts response to tamoxifen in elderly women with breast cancer. *Ann. R. Coll. Surg. Engl.*, **73**, 361-363.

DIAMOND SL, SHAREFKIN JB, DIEFFENBACH C, FRASIER-SCOTT K, MCINTIRE LV AND ESKIN SG. (1990). Tissue plasminogen activator messenger RNA levels increase in cultured human endothelial cells exposed to laminar shear stress. *J. Cell. Physiol.*, **143**, 1757-1761.

DIXON JM. (1992). Treatment of elderly patients with breast cancer-tamoxifen alone is no longer justified. *Br. Med. J.*, **304**, 996-997.

EARLY BREAST CANCER TRIALISTS' COLLABORATIVE GROUP. (1992). Systemic treatment of early breast cancer by hormonal, cytotoxic, or immune therapy. *Lancet*, **339**, 1-15.

FLOWERS JL, BURTON GV, COX EB, MCCARTY KS, DENT GA, GEISINGER KR AND MCCARTY KS. (1985). Use of monoclonal antiestrogen receptor antibody to evaluate estrogen receptor content in fine needle aspiration breast biopsies. *Ann. Surg.*, **203**, 250-254.

GAZET, J-C AND FORD HT. (1987). Prediction of endocrine response in breast cancer by immunocytochemical detection of oestrogen receptor in fine-needle aspirates. *Lancet*, **2**, 701-703.

FUQUA SAW, FALETTE NF AND MCGUIRE WL. (1990). Sensitive detection of estrogen receptor RNA by polymerase chain reaction. *J. Nat. Cancer Inst.*, **82**, 858-861.

GASKELL DJ, SANGSTERI K, HAWKINS RA AND CHETTY U. (1989). Relation between immunocytochemical estimation of oestrogen receptor in elderly patients with breast cancer and response to tamoxifen. *Lancet*, **2**, 1044-1046.

GAZET JC, MARKOPOULOS C, FORD HT, COOMBES RC, BLAND JM & DIXON RC. (1988). Prospective randomised trial of tamoxifen versus surgery in elderly patients with breast cancer. *Lancet*, **2**, 679-681.

GREEN S, WALTER P, KUMAR V, KRUST A, BORNERT J-M, ARGOS P AND CHAMBON P. (1986). Human oestrogen receptor cDNA: sequence, expression and homology to v-erb-A. *Nature*, **320**, 134-139.

HENRY JA, PIGGOTT NH, MALLICK UK, NICHOLSON S, FARNDON JR, WESTLEY BR AND MAY FEB. (1991). pNR-2/pS2 immunohistochemical staining in breast cancer: correlation with prognostic factors and endocrine response. *Br. J. Cancer*, **63**, 615-622.

HOOF T, RIODAN JR AND TÜMMLER B. (1991). Quantitation of mRNA by the kinetic polymerase chain reaction: a tool for monitoring P-glycoprotein gene expression. *Anal. Biochem.*, **196**, 161-169.

HORWITZ KB AND MCGUIRE WL. (1978). Estrogen control of progesterone receptor in human breast cancer. *J. Biol. Chem.*, **253**, 2223-2228.

JAKOWLEW SB, BREATHNACH R, JELTSCH J-M, MASIAKOWSKI P AND CHAMBON P. (1984). Sequence of the pS2 mRNA induced by oestrogens in the human breast cancer cell line MCF-7. *Nucleic Acids Res.*, **12**, 2861-2878.

JOHNSON MD, WESTLEY BR AND MAY FEB. (1989). Oestrogenic activity of tamoxifen and its metabolites on gene regulation and cell proliferation in MCF-7 breast cancer cells. *Br. J. Cancer*, **59**, 727-738.

KASTNER P, KRUST A, TURCOTTE B, STROPP U, TORA L, GRONEMEYER H AND CHAMBON P. (1990). Two distinct estrogen-regulated promoters generate transcripts encoding the two functionally different human progesterone receptor forms A and B. *EMBO J.*, **9**, 1603-1614.

KUNKEL TA, ROBERTS JD AND ZAKOUR RA. (1987). Rapid and efficient site specific mutagenesis without phenotypic selection. *Methods Enzymol.*, **154**, 367-382.

MCGUIRE WL, CARBONNE PD AND VOLLMER RP (EDS). (1975). *Estrogen Receptor and Human Breast Cancer*. Raven Press: New York.

MANNI A. (1989). Endocrine therapy of metastatic breast cancer. *J. Endocrinol. Invest.*, **12**, 357-372.

MAY FEB AND WESTLEY BR. (1986). Cloning of estrogen-regulated messenger RNA sequences from human breast cancer cells. *Cancer Res.*, **46**, 6034-6040.

MAY FEB AND WESTLEY BR. (1987). Effects of tamoxifen and 4'-hydroxytamoxifen on the pNR-1 and pNR-2 estrogen-regulated genes from the MCF-7 human breast cancer cell line. *J. Biol. Chem.*, **262**, 15894-15899.

MAY FEB AND WESTLEY BR. (1988). Identification and characterisation of estrogen-regulated RNAs in human breast cancer cells. *J. Biol. Chem.*, **263**, 12901-12908.

MAY FEB, JOHNSON MD, WISEMAN LR, WAKELING AE, KASTNER P AND WESTLEY BR. (1989). Regulation of progesterone receptor mRNA by oestradiol and antioestrogens in breast cancer cell lines. *J. Steroid Biochem.*, **33**, 1035-1041.

OSBORNE CK, YOCHMOWITZ MG, KNIGHT WA AND MCGUIRE WL. (1980). The value of oestrogen and progesterone receptors in the treatment of breast cancer. *Cancer*, **46**, 2884-2888.

PIGGOTT NH, HENRY JA, MAY FEB AND WESTLEY BR. (1991). Antipeptide antibodies against the pNR-2 oestrogen-regulated protein of human breast cancer cells and detection of pNR-2 expression in normal tissues by immunohistochemistry. *J. Pathol.*, **163**, 95-104.

POULSOM R AND WRIGHT NA. (1993). Trefoil peptides: a newly recognized family of epithelial mucin-associated molecules. *Am. J. Physiol.*, **265**, 205-213.

RAPPOLEE DA, MARK D, BANDA MJ AND WERB Z. (1988). Wound macrophages express TGF-α and other growth factors in vivo: analysis by mRNA phenotyping. *Science*, **241**, 708-712.

RAPPOLEE DA, WANG A, MARK D AND WERB Z. (1989). Novel method for studying mRNA phenotypes in single or small numbers of cells. *J. Cell. Biochem.*, **39**, 1-11.

SIEBERT PD AND LARRICK JW. (1992). Competitive PCR. *Nature*, **359**, 557-558.

WESTLEY BR, HÖLZEL F AND MAY FEB. (1989). Effects of oestrogens and the antioestrogens, tamoxifen and LY117018, on four oestrogen-regulated RNAs in the EFM-19 breast cancer cell line. *J. Steroid Biochem.*, **32**, 365-372.

WILSON YG, RHODES M, IBRAHIM NBN, PADFIELD CJH AND CAWTHORN SJ. (1994). Immunocytochemical staining of pS2 protein in fine-needle aspirate from breast cancer is an accurate guide to response to tamoxifen in patients over 70 years. *Br. J. Surg.*, **81**, 1155-1158.

WUNDRACK I, MÜLLENBACH R, WELTERS C, SEITZ G AND BLIN N. (1994). PCR expression analysis of the estrogen-inducible gene BCEI in gastrointestinal and other human tumors. *Disease Markers*, **12**, 63-69.